# Contrasting scaling properties of interglacial and glacial climates

**DOI:** 10.1038/ncomms10951

**Published:** 2016-03-16

**Authors:** Zhi-Gang Shao, Peter D. Ditlevsen

**Affiliations:** 1 Guangdong Provincial Key Laboratory of Quantum Engineering and Quantum Materials, SPTE, South China Normal University, Guangzhou 510006, China; 2 Centre for Ice and Climate, Niels Bohr Institute, University of Copenhagen, Juliane Maries Vej 30, Copenhagen 2100, Denmark

## Abstract

Understanding natural climate variability is essential for assessments of climate change. This is reflected in the scaling properties of climate records. The scaling exponents of the interglacial and the glacial climates are fundamentally different. The Holocene record is monofractal, with a scaling exponent *H*∼0.7. On the contrary, the glacial record is multifractal, with a significantly higher scaling exponent *H*∼1.2, indicating a longer persistence time and stronger nonlinearities in the glacial climate. The glacial climate is dominated by the strong multi-millennial Dansgaard–Oeschger (DO) events influencing the long-time correlation. However, by separately analysing the last glacial maximum lacking DO events, here we find the same scaling for that period as for the full glacial period. The unbroken scaling thus indicates that the DO events are part of the natural variability and not externally triggered. At glacial time scales, there is a scale break to a trivial scaling, contrasting the DO events from the similarly saw-tooth-shaped glacial cycles.

## Abstract (web summary)

Natural climate variability and persistency are reflected in the scaling properties of climate records. Here, the authors show that the scaling properties of interglacial and glacial climates are distinctively different: The former is monofractal while the latter is multifractal with much longer range memory.

## Graphical abstract

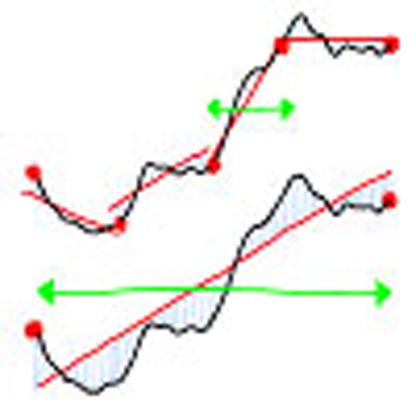

The climate system is characterized by complex interactions between atmosphere, oceans, ice, landmasses and the biosphere over a large range of temporal and spatial scales. For understanding natural climate variability and the character of climate change, assessing correlation and persistence times is important. These are reflected in the scaling properties of the climatic records. Scaling was first noted in the seminal work by Hurst^1^ on reservoir capacity and runoff in the Nile. Hereafter, it was realized that time series of complex systems such as the climate system are characterized by fractal^2^ or even multifractal^3^ scaling properties. It has been a long-standing discussion to which extent the fractal nature of the climate dynamics is universal or if it is more specific to the processes and range of scales observed^3^,^4^,^5^,^6^.

To identify the underlying dynamics reflected in scale breaks or robust scaling relations, records covering a large range of temporal scales are necessary. On the atmospheric variability, a range of instrumental records of temperatures and other meteorological parameters have been investigated^3^,^4^,^7^. On short time scales the turbulent nature of the atmospheric fields is measured in airplane and drop-sonde campaigns. From these measurements, multifractal scaling of the fields has been reported^8^.

On longer time scales, instrumental records exist for ∼150 years, typically with daily or twice daily resolution. By filtering out the annual cycle, the scaling properties of temperature variations, covering four to five decades, have been investigated. These indicate universal persistence laws for atmospheric variability^4^, where station data from around the globe shows monofractal (Hurst)-scaling exponents around 0.7. This is significantly different from the value 0.5 characteristic for a trivial white noise process. On even longer time scales we rely on proxy records, where the ice core records are especially suited, as they can be understood as high-resolution sedimentation records from the atmosphere. It was in ice core records that it was first realized that the glacial climate was dominated by millennial scale instabilities, the Dansgaard–Oeschger (DO) events^9^. These events occur all the way from the last inception through to the termination of the last glacial period. One striking feature although is that in the period around the last glacial maximum (LGM, 27–15 kyr) the record only contains a single small event (DO2).

Here we find, by analysing climate periods separately, that the Holocene and the glacial climates have distinctly different scaling properties. The Holocene is monofractal with a scaling exponent *H*∼0.7, whereas the glacial climate is multifractal with *H*_2_∼1.2. The longer persistence time in the glacial period is expected, owing to the presence of the pronounced millennium scale DO events. However, the same scaling is found for the LGM period, indicating that the DO events are not the cause and should be seen as an intrinsic part of the glacial climate. The DO events have a characteristic saw-tooth shape in the records, with rapid warming and slow cooling, similar to the shape of the externally forced glacial cycles with fast terminations and slow inceptions. By analysing the much longer 5 Myr ocean sediment climate record^10^ and the 800-kyr Antarctic EPICA (European Project for Ice Coring in Antarctica) ice core record^11^, we find a scale break around 20 kyr, such that on glacial time scales (>20 kyr) we have a trivial scaling *H*=0.5. We might speculate that this reflects that DO events are internally generated, whereas glacial cycles are externally forced. It is noteworthy that for a trivial red noise process, the so-called Ornstein–Uhlenbeck process, the scaling spectrum shows a cross-over from *H*=3/2 for time scales shorter than the correlation time to *H*=1/2 for time scales longer than the correlation time. The cross-over time scale indicates the internal time scale of built-up of the large ice sheets.

## Results

### The climate record

To determine to which extent the scaling behaviour extends to scales beyond the length of the instrumental temperature records, we thus rely on paleoclimatic proxies. The stable isotope (*δ*^18^*O* and *δD*) records obtained from the Greenland and Antarctic ice sheets constitute such temperature proxies, with a sufficiently linear relationship to the average atmospheric temperature mixed with an independent noise, which the scaling properties of the atmospheric temperature can be assumed to be reflected in the record^12^.

**Figure 1:**
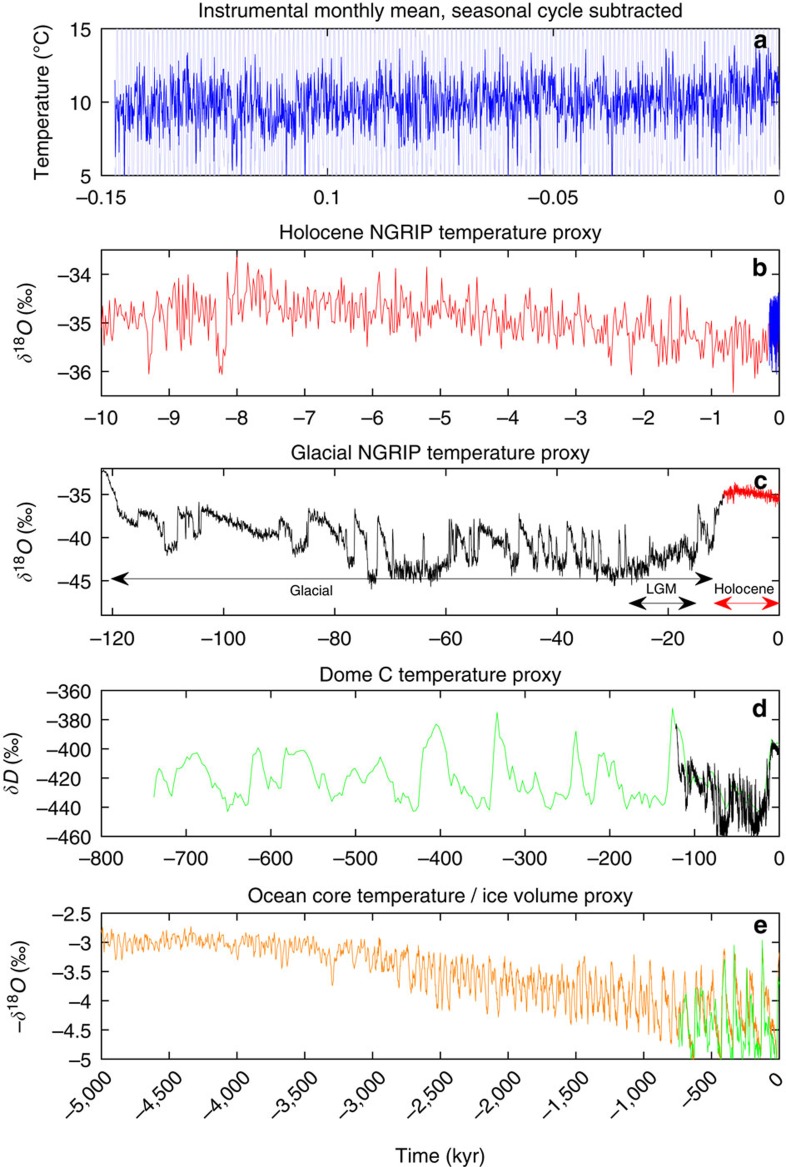
Temperature variations from monthly to geological time scales. (**a**) A European meteorological station record (http://www.metoffice.gov.uk/pub/data/weather/uk/climate/stationdata/oxforddata.txt (accessed 2015). URL http://www.metoffice.gov.uk/pub/data/weather/uk/climate/stationdata/oxforddata.txt). ranging over 150 years (light blue, full range not shown). The record with the seasonal cycle subtracted is shown in blue. (**b**) The Holocene part of the NGRIP isotope record^6^. The instrumental record normalized to the ice core record (arbitrary) is shown in blue. (**c**) The full NGRIP record, dated using the ‘GICC05modelext' chronology. The *δ*^18^*O* is a linear proxy for temperature. The warm Holocene period 11.7 kyr to present (red, corresponding to plot (**b**)) is remarkably stable in comparison with the previous glacial period 12–120 kyr B2K (long black arrow). Before that the end of the previous warm period (Eem) is seen. LGM (15–27 kyr B2K) experienced only one small DO event. (**d**) The Antarctic EPICA *δD* record^21^ spanning almost eight glacial cycles at 3 kyr resolution. The black curve covering the last glacial period is the (normalized) record shown in **c**. (**e**) The stacked marine benthic foraminiferal isotope record^16^ (minus). This is a proxy for global ice volume and global deep ocean temperature. In green is the (normalized) curve in **d**.

The issue of nonlinearity and multifractality on multi-glacial cycle time scales has been addressed before in the analysis of the Antarctic Vostok record^13^ and comparison with Greenland Icecore Project (GRIP), Greenland Ice Sheet Project (GISP) (Greenland) and Taylor dome (Antarctica) ice core records. The analysis show slightly different scaling exponents between the different ice core records, indicating that dating accuracy and period analysed are important for the results (GRIP and GISP cores should, due to close proximity, give the same result). The influence of chronology on the scaling properties is confirmed by an analysis of the North Greenland Icecore Project (NGRIP) record^14^,^15^ (Fig. 1c), where the scaling properties of the recent 2,000-year *δ*^18^*O* record is different from the properties of the record older than 2,000 years^16^. This should be expected, as the climate is influenced by different processes operating at different time scales. The NGRIP ice core^14^ has been dated with unprecedented accuracy over the past 122 kyr (ref. 15) This enables us to accurately calculate the scaling properties for the different climate states separately.

On multi-millennial time scales, it is known that glacial cycles are linked, in a nonlinear manner^13^, to periodic and quasi-periodic changes in the insolation (incoming solar radiation) from variations in Earth's orbit around the Sun. The climate response is ∼100 kyr glacial cycles since the middle Pleistocene transition around 1 Myr ago, where the climate has shifted regularly between the glacial and interglacial climate states.

The temperature and proxy records ranging from the instrumental record to five million years, obtained from the stacked deep ocean sediment record^10^, over a huge range of scales are shown in Fig. 1. As there is a connection between spatial and temporal scales, the records shown will be more local in the top panels and more global in the bottom panels. The Greenland ice core record represent a composite of a local and a Northern Atlantic climate signal. To eliminate the influence of long-term trends on the scaling properties, we employ the multifractal detrended fluctuation analysis (MF-DFA) method^17^ for the analysis of the records. We have employed the DFA analysis of both first and second order (DFA1 and DFA2), and found that our results are robust in the sense that there is very little difference between the two for the analysed data (see Supplementary Figs 1 and 2). Here we report for DFA1 (see Methods section for description of the MF-DFA). The MF-DFA is complementary to a power spectrum analysis, where the discrete components such as the diurnal and seasonal cycle, as well as the orbital periods on Milankovitch timescales, are mixed with the continuous part of the spectrum^18^,^19^. If the continuous spectrum scales with frequency *P*(*f*)∼*f*^−*β*^, there is a direct linear relation between the scaling exponents for the spectrum and for the fluctuations^20^; *H*=(*β*+1)/2. Thus, the trivial white noise power spectrum *β*=0 corresponds to *H*=0.5, whereas the trivial red noise spectrum *β*=2 corresponds to *H*=1.5. This simply follows from the power spectrum being the Fourier transform of the autocorrelation function. The multifractality is not captured in the powerspectrum.

### Multifractal detrended fluctuation analysis

**Figure 2:**
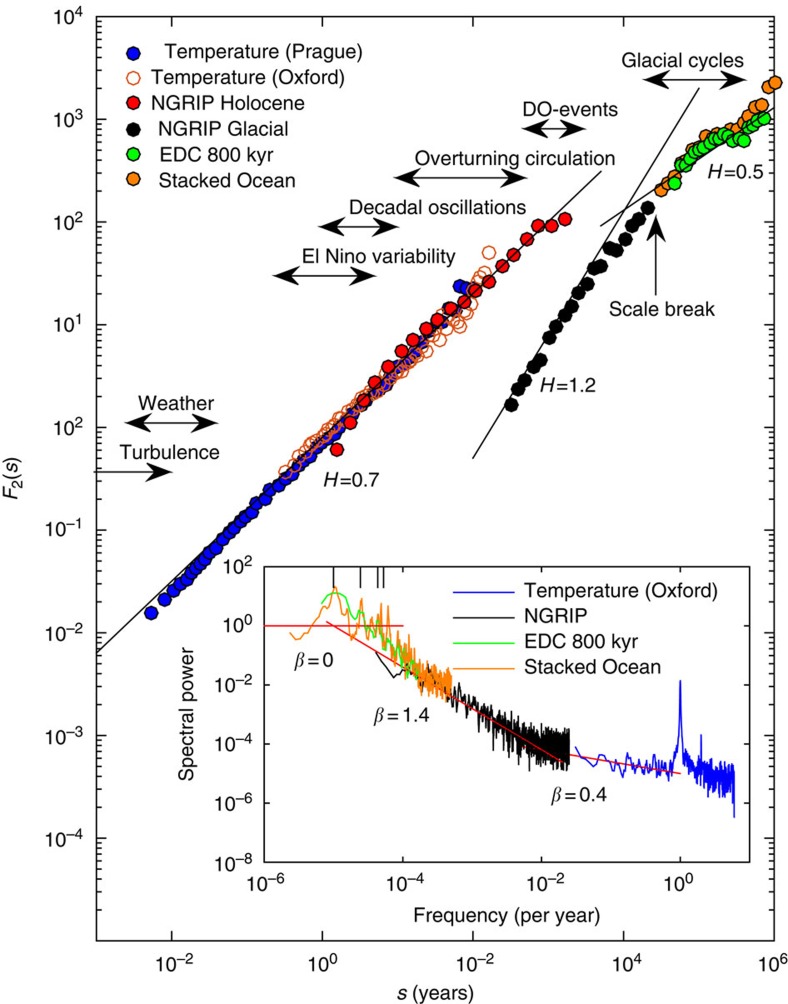
The mean variance in a time window scale with the length of the window. Main panel shows the fractal spectrum *F*_2_(*s*) of the climate records. The scaling for the Holocene range from days to millennia (blue and red dots). The scaling exponent *H*=*H*_2_ is the slope of the line. The typical time scales of different processes in the climate system are indicated. The scale break around 20 kyr is noteworthy. The insert shows the power spectra of the climatic time series (same colours). The slopes of the continuous part of the spectra corresponds to the (monofractal) Hurst exponent through *H*=(*β*+1)/2. The power spectra mix the continuous (scaling) part and the discrete peaks corresponding to periodic and quasi-periodic components. The small black bars in the top left corner of the insert indicates the Milankovitch periods at 19, 23, 41 and 100 kyrs.

**Figure 3:**
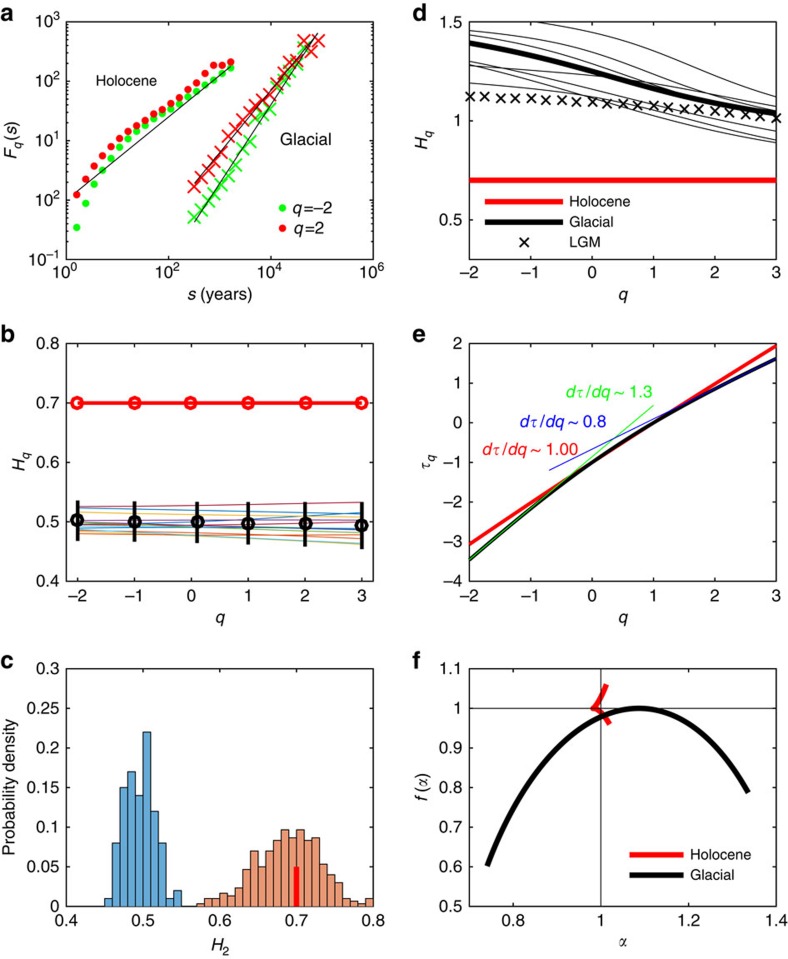
Contrasting monofractal scaling for Holocene and multifractal scaling for glacial periods. The scaling spectra *F*_*q*_(*s*) for the Holocene and glacial periods are shown in (**a**). Holocene shows monofractal scaling, whereas the glacial is multifractal, as there is a significant difference in the slopes for *q*=−2 and *q*=2. (**b**) The scaling in a set of 100 reshuffled versions of the record (thin lines, not all shown). The black dots are the means for the reshuffled data (true value is 0.5) and the bars are the 2*σ* spreads. (**c**) The distribution of Hurst exponent *H*_2_ for the 100 reshuffled series, centred around *H*_2_=0.5 (blue histogram). The Holocene record is marked by the red bar. The orange histogram is the distribution for a set of 100 simulations of fractional Brownian motion with *H*_2_=0.7. (**d**) The generalized Hurst exponent *H*_*q*_ is shown for Holocene (red, same as in **b**) and the glacial period (thick black). The thin black curves are for non-overlapping 12 kyr sections of the glacial periods, indicating the range of uncertainty. The black crosses is the LGM, where only one short DO event occurs. (**e**) The multifractal scaling exponent 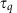 for Holocene (red) and the glacial period (black), where a change in slope from negative (green line) to positive (blue line) values of *q* is observed. The Holocene curve is indistinguishable from a straight line with slope 1.00. (**f**) The multifractal spectrum *f*(*α*) versus 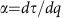. The small red wedge indicates that the Holocene record is almost monofractal (for which the spectrum (*α*, *f*(*α*)) would collapse to the point (1, 1). This is also seen in the perfect linear fit in **b**. The black curve is for the glacial record, indicating strong multifractality.

We first analyse the Holocene climate (0–11.7 kyr B2K). The scaling of the Holocene represented by the ice core compares well with the instrumental record (Fig. 2); this shows a remarkable range of scaling over more than five decades, from a day to a few thousand years. In the insert, the corresponding power spectra are shown. Here the pronounced spectral peaks, the year and the Milankovitch periods, are mixed with the continuous part of the spectra. The Holocene climate shows monofractal scaling, with a scaling exponent *H*∼0.7, significantly different from the trivial value *H*=0.5. Figure 3a shows the spectra *F*_*q*_(*s*) for *q*=±2, for both Holocene and the glacial records. For Holocene, the black line corresponds to *H*=0.7, which fits for both values of *q*. This is in contrast to the glacial record that shows multifractal scaling with *H*_−2_=1.4 and *H*_2_=1.2. The Holocene record is tested against a Monte Carlo reshuffling, which preserved the probability density of the data, see Fig. 3b and blue histogram in Fig. 3c. We have also tested the record against an autoregressive process with identical lack-one autocorrelation, with similar results (not shown). To further investigate the reliability, we have generated time series of same length as the record from a fractional Brownian motion, with Hurst exponent 0.7 (Supplementary Figs 3 and 4). The probability density of measured exponents is shown in Fig. 3c (orange histogram). It is noteworthy that the consistency with the record (red bar) is a much weaker result than rejection of the null hypothesis above. It indicates the uncertainty in the estimated scaling exponent due to the limited length of the record. As we do not have a full theory of the underlying climate processes generating the fractal structure, we cannot rule out that the Holocene is weakly multifractal but the time series is to short to detect the multifractality. To obtain some indications on this possibility, we have simulated a known weakly multifractal process of similar length to the record (Supplementary Figs 5–7), where the multifractallity can indeed be detected. This we interpret as further supporting the observation that the Holocene record is monofractal.

The findings for the Holocene is in agreement with findings from climate model millennial simulations^21^. In Supplementary Fig. 8 we present an analysis of two of the CESM1-CAM5 Last Millennium Ensemble runs.

For longer time scales we enter the glacial climate state and on even longer time scales we observe a scale break at the Milankovitch time scales (>20 kyr). Analysis of the 800-kyr Antarctic Epica Dome C (EDC) core^11^ with 3 kyr resolution and the 5.3-Myr stacked ocean benthic foraminiferal isotope record^10^ (green and orange curves in Fig. 1d,e) does indeed show that these records have a trivial scaling with a Hurst exponent close to 0.5 (green and orange dots in Fig. 2). As a further consolidation of the robustness we also analysed GRIP ice core on the GICC05 time scale, in agreement with the results for NGRIP (Supplementary figs 9 and 10). Furthermore, we have analysed the 420-kyr Vostok core^22^, which agreed with the results for EDC (not shown).

**Figure 4:**
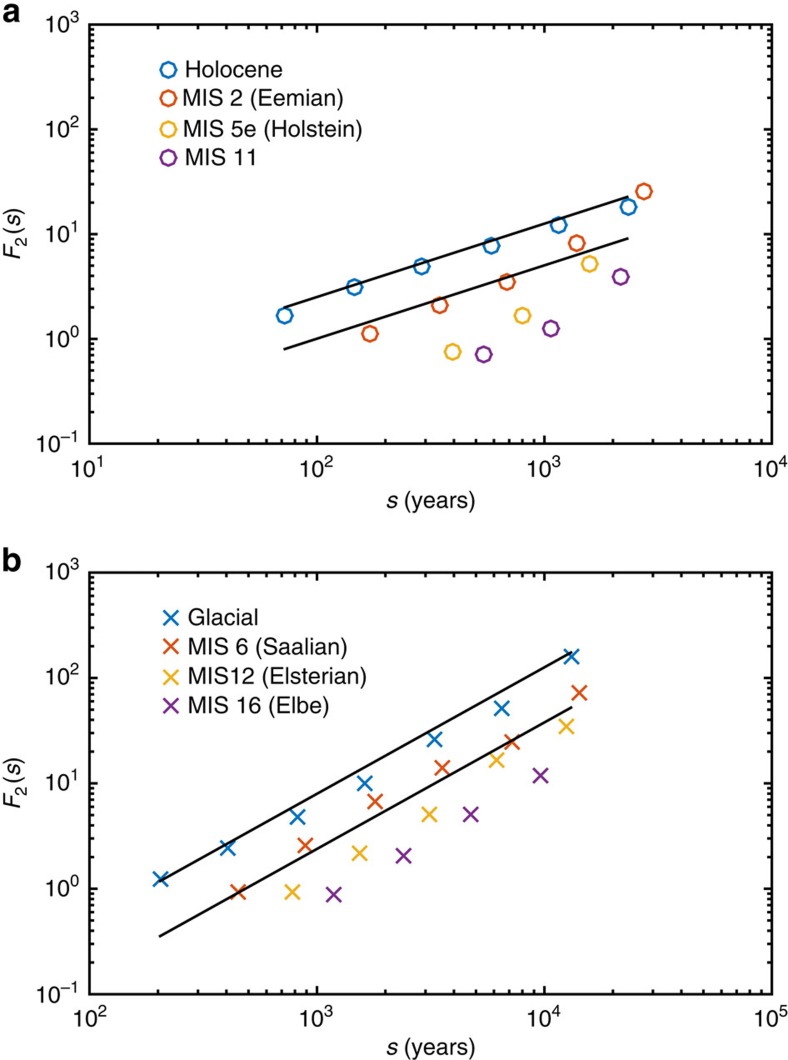
Comparing scaling for interglacial and glacial periods. The scaling spectra *F*_2_(*s*) for the EDC core split into (**a**) interglacial periods: Holocene (0–12 kyr BP), MIS 2 (113–130 kyr BP), MIS 5e (235–243 kyr BP) and MIS 11 (321–335 kyr BP); and (**b**) glacial periods: last glacial (16–113 kyr BP), MIS 6 (136–235 kyr BP), MIS 12 (246–321 kyr BP) and MIS 16 (340-396 kys BP). The straight lines have slopes *H*_2_=0.7 for interglacials and *H*_2_=1.2 for glacials.

The ice core record (Fig. 1c) shows that the warmer climate of the Holocene period (0–11.7 kyr B2K) is more stable than that of the last glaciation (12–120 kyr B2K)^23^. The difference in climate states is reflected in the scaling properties of the proxy temperature signal; thus, we split the NGRIP *δ*^18^*O* signal into two parts covering the Holocene warm period and the last glacial period. The glacial period was characterized by millennium scale sudden climate shifts, the DO events^9^, the cause of which is still unknown. The predominant assumption for the cause of the DO events is abrupt changes in the Atlantic meridional overturning circulation^24^,^25^ perhaps triggered by (negative) changes in freshwater forcing. Many mechanisms have been proposed as a trigger, from oscillations in the ice sheets^26^, ice shelf breakup^27^ or sea ice switching^28^, or changes in solar output^29^. The occurrence of DO events influences the correlation time and thus the scaling properties of the record. One could speculate that the larger glacial Hurst exponent is a consequence of the presence of the DO events alone. This is not the case, as the LGM period, 15–27 kyr B2K, with only one short DO event (DO2) does also show a scaling, which is significantly different from the Holocene climate (black crosses in Fig. 3d). To assess the robustness, we furthermore analyse non-overlapping 12 kyr glacial periods (same duration as the Holocene). For all these periods and for the full glacial period, we get multifractal scaling with *H*=*H*_2_∼1.2 (thin black lines in Fig. 3d). As the DO events are a part of the glacial record scaling, this indicates that they are part of the internal variability and not externally caused, in contrast to the glacial cycles, which are forced by the Milankovitch cycles and show trivial scaling. Confirming the robustness of the results is rather delicate, as there is only a limited set of truly independent paleoclimatic records, each influenced by independent noise processes, which might mask the scaling properties inferred for the climate. One possible test of the observed difference between the interglacial and the glacial climate is to investigate the previous interglacial and glacial periods separately. This we have done by splitting the EDC record into interglacial and glacial periods, and analysing them separately. The result is shown in Fig. 4 and are consistent with the results found for the Greenland ice cores. Furthermore, we have confirmed the results by repeating the same analysis for the EDML and Vostok cores (Supplementary Figs 11 and 12).

The Greenland stable isotope ice core records are proxies for some large-scale North Atlantic mean temperature. Nearby North Atlantic sea surface temperatures has been reconstructed for the Holocene from ocean sediment cores^30^,^31^ Despite proximity to the Greenland ice core sites, these show much more variability in the Holocene climate. The scaling exponents are *H*∼1.1 for LO09-14 from the North Atlantic current at the Reykjanes Ridge south of Iceland and *H*∼1.4 for MD95-2011 in the Norwegian Atlantic current (Supplementary Fig. 13). The two records are slightly anticorrelated (corr=−0.25), indicating that they locally monitor fractions of the northward Atlantic heat transport, which can be much more variable than the mean climate of the North Atlantic.

To investigate further their multifractality and difference, Fig. 3e shows the multifractal scaling exponent 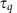 (see Methods section) for Holocene and the glacial period, where a change in slope from negative to positive values of *q* is observed. This means that there is an asymmetry in the scaling properties of large and small fluctuations. This has been quoted a multifractal phase transition^32^. In multifractal phase transition, two critical *q* orders defined as *q*_*s*+_ and *q*_*s*−_ exist^16^. When *q*>*q*_*s*+_ or *q*<*q*_*s*−_, 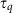 will be linear as a function of *q*, as the largest fluctuations dominate the empirical moments^6^. We observe that *q*_*s*+_≈3 and *q*_*s*−_≈−2 for the glacial record; thus, we can limit the plotting interval for *q* from −2 to 3 in Fig. 3e. Figure 3f shows the multifractal spectrum *f*(*α*) versus 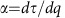. For monofractals the (*α*, *f*(*α*)) spectrum collapses to the point (1, 1). The small red wedge indicates that the Holocene record is monofractal, whereas the black curve shows strong multifractality in the glacial climate.

### The tail of the probability distribution

**Figure 5:**
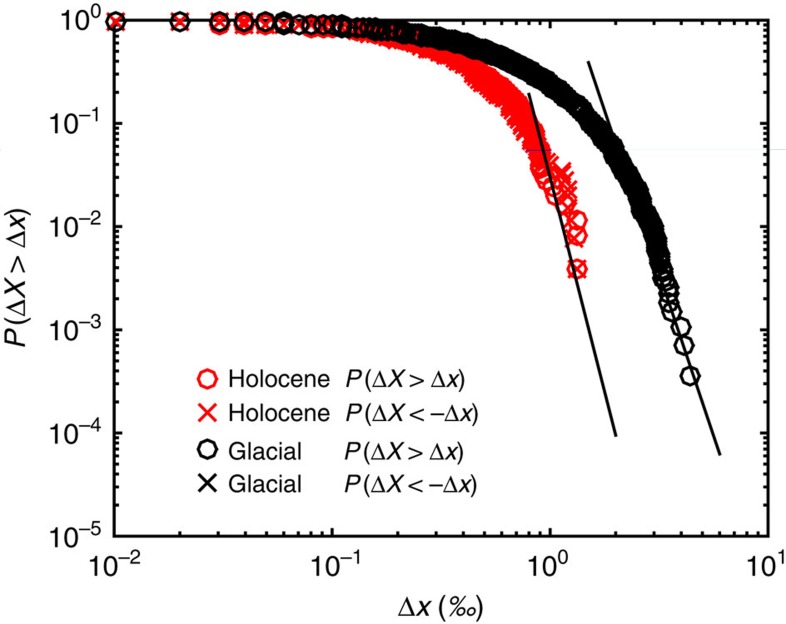
Comparing the extreme tails of the distributions for Holocene and glacial records. Comparison of probability distribution *Pr* (Δ*X*>Δ*x*) versus Δ*x* between the Holocene (red) and last glacial (black) records. Δ*x*_*t*_=*x* (*t*+20yrs)−*x* (*t*), where *x* (*t*) is the *δ*^18^*O* record of NGRIP ice core. For both parts of the record, the extreme tail is a power law, *P*(Δ*X*′>Δ)∼Δ^*γ*^, represented by the straight lines with slopes *γ*≈−8.3 (Holocene) and *γ*≈−6.3 (glacial).

The difference of multifractality in the records of the two periods reflects that the climate of the interglacial is quite different from the glacial climate: The DO events introduce long-range correlations related to the waiting times of several thousand years for jumping between the stadial and the interstadial states. This jumping between states is absent in the Holocene climate. As the DO events do not lead to a scale break in the scaling of the glacial climate signal, we speculate that this is an indication that they do not have a trigger, which is disconnected (such as changing solar radiation) from the climate dynamics giving rise to the scaling properties. The occurrence of the DO events seems random in nature^33^, which agrees well with internal fluctuations in the Atlantic meridional overturning circulation as the cause of these events. The reason why DO variability is absent in the Holocene climate could be attributed to the absence of the large ice sheets. However, this does not necessarily imply ice sheet instability to be the trigger. It could well be that the larger short-term variability in the glacial climate strongly enhance the triggering. The short-term fluctuations can be represented by Δ*x*_*t*_=*x*(*t*+Δ*t*)−*x*(*t*), where *x*(*t*) is the given evenly spaced time series. A generic character of multifractal processes is that they have fat-tailed probability distributions, that is, *P* (Δ*X*>Δ*x*)∼(Δ*x*)^−*γ*^, for large Δ*x* (and similar for the negative tail: *P* (Δ*X*<−Δ*x*)), where *γ* is the corresponding probability exponent^6^. Figure 5 shows the comparison of probability distributions *P* (|Δ*X*|>Δ) versus Δ*x* for the the Holocene (red) and last glacial (black) (positive and negative tails have identical distributions). The sizes of the fluctuations are larger in the glacial period but interestingly the probability distributions for both the Holocene and the glacial show fat-tail scaling (straight black line) for large Δ*x* with *γ*≈−8.3 (Holocene) and *γ*≈−6.3 (glacial). One could speculate that the less extreme climate of the Holocene prevents triggering of DO events.

## Discussion

In summary, the interglacial climate shows scaling over a remarkable range of scales from daily to millennial. The generalized Hurst exponent of *H*∼0.7 is significantly different from the trivial value *H*=0.5. The glacial climate state has a distinctly more fractal characteristics, with a much larger generalized Hurst exponent *H*∼1.2. Although neither the Antarctic- nor the Greenland ice core records represent a global climate signal, the differences in scaling exponents reported for different records^6^ in the range 1.2–1.4 are in our judgement identical within the uncertainty of measurement.

The glacial record also shows a clear multifractal scaling, with an asymmetry between small and large fluctuations. The DO events are a part of the scaling process, indicating that they are part of the internal variability, and not externally caused, in contrast to the glacial cycles, which are forced by the Milankovitch cycles and show trivial scaling.

## Methods

### Multifractal detrended fluctuation analysis

The MF-DFA^17^ is a robust and easily implemented analysis of the scaling properties in strongly fluctuating or non-stationary time series. It is performed in five steps as follows: (i) determine the cumulated data series


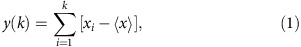


where 〈*x*〉 is the mean value of the time series *x*_*k*_ (*k*=1,...,N). (ii) The profile is divided into N_*s*_=int(N/*s*) disjoint segments with same size *s*. As the congruence between N and *s* is often not zero, a part will remain after division. To preserve this part, the same dividing procedure is repeated from the opposite end. As a consequence, 2*N*_*s*_ segments are generated. (iii) The variance is calculated as


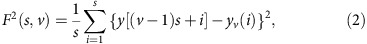


where *v*=1, …, N_*s*_ and


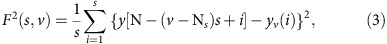


where *v*=N_*s*_+1,..., 2N_*s*_. *y*_*v*_(*i*) is the least square-fitting line in segment *v*. Next, the *q*-th order fluctuation function is


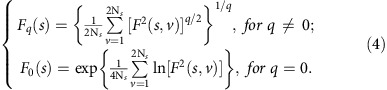


(iv) The above steps (ii) and (iii) are repeated as the segment size *s* increases. (v) The scaling exponent is determined by fitting log–log plots of *F*_*q*_(*s*) versus *s* as


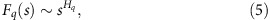


where *H*_*q*_ is the generalized Hurst exponent. Next, the multifractal spectrum (*f*(*α*) versus *α*) can be derived using the following relationship^16^,^17^:


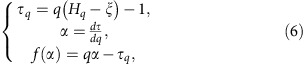


where *α* is the singularity strength, 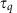 is the multifractal scaling exponent and *f* (*α*) is the dimension as a function of the *α*. *ξ*=*H*_1_−1 is the scaling exponent of the mean fluctuations. For monofractal time series, the (*α*, *f*(*α*)) spectrum collapses to the point (1, 1). In practice, for finite time series *f* (*α*) versus *α* will be a tiny arc solely due to random fluctuation. For multifractal time series, the multifractal spectrum will typically have a single-humped parabolic shape^17^.

## Additional information

**How to cite this article:** Shao, Z.-G. & P. D. Ditlevsen. Contrasting scaling properties of interglacial and glacial climates. *Nat. Commun.* 7:10951 doi: 10.1038/ncomms10951 (2016).

## Supplementary Material

Supplementary Information Supplementary Figures 1-13 and Supplementary References
