# Supplementary material for: Contrasting scaling properties of interglacial and glacial climates
Source: Nat Commun. 2016 Mar 16;7:10951. doi: 10.1038/ncomms10951 (PMC4799372; doi:10.1038/ncomms10951)
Supplement: Supplementary Information — Supplementary Figures 1-13 and Supplementary References [file ncomms10951-s1.pdf]

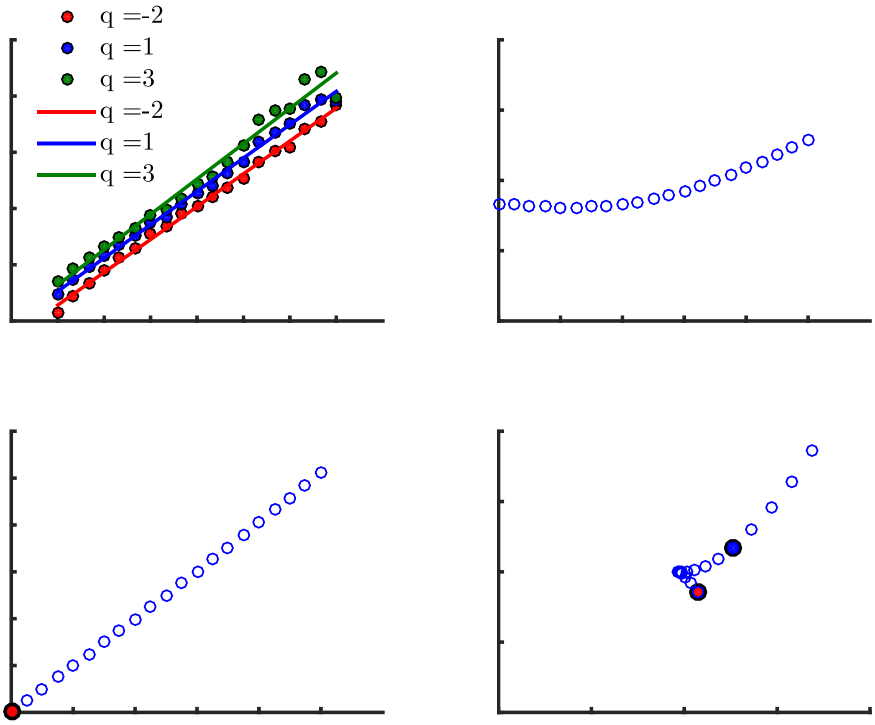

Supplementary Figure 1. Order of MF-DFA. The order of the DFA analysis signifies the degree of the polynomial subtracted from the time series before calculating the variance, thus for DFA1 only the trend is removed, while for DFA2 the best fit second order polynomial (within the specific window) is subtracted. For analysis of a process of unknown origin there are no theoretical arguments for choosing a specific order. Thus we should be concerned if the order chosen for analysis does change the results in any significant matter. Here the MF-DFA1 for the NGRIP Holocene record is shown.

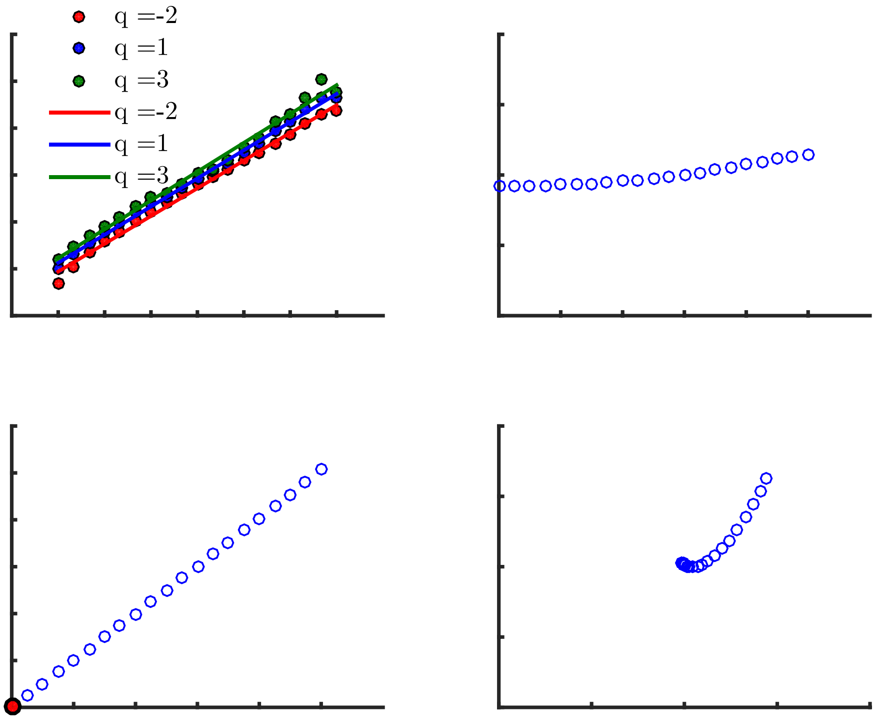

Supplementary Figure 2. Second order MF-DFA. The MF-DFA2 analysis for the NGRIP Holocene record is almost identical to the MF-DFA1.

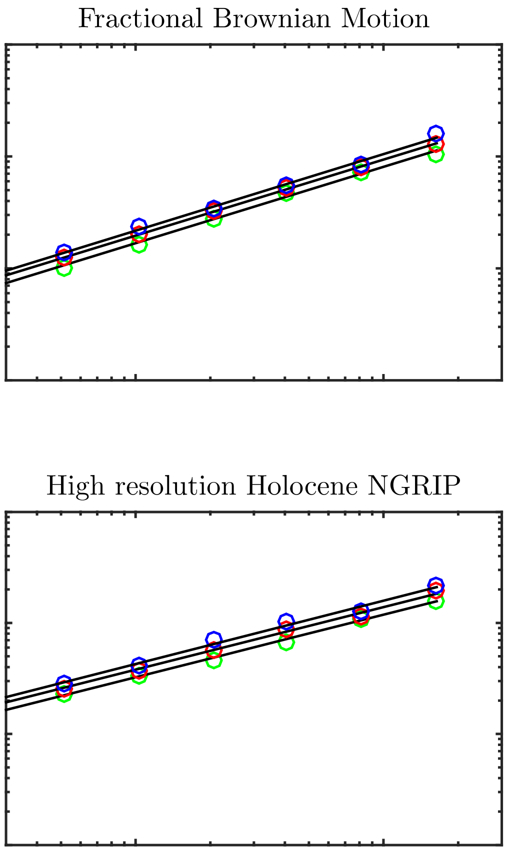

Supplementary Figure 3. Estimating uncertainty in scaling. The problem of estimating uncertainties is two-fold. Firstly, for a limited time series (data) of an unknown process it can of course not be established that it is truly scaling. Since for any realisation the scaling will not be prefect, it can only be established that the scaling of the data is consistent with scaling of similar simulated realisations from a known scaling process. Secondly, assuming such an underlying process, we can estimate the uncertainty in the scaling exponent by defining the observed scaling exponent as the maximum likelihood exponent for the known process, and then by simulation establish the uncertainty in estimating the (known) exponent from a realisation of a given length.
Shown are the spectra of the FBM and the Holocene records, the scattering around the perfect scaling lines for the Holocene record is comparable to the scattering for a realization of the fractional brownian motion with the same record length and the scaling exponent derived from the Holocene record.

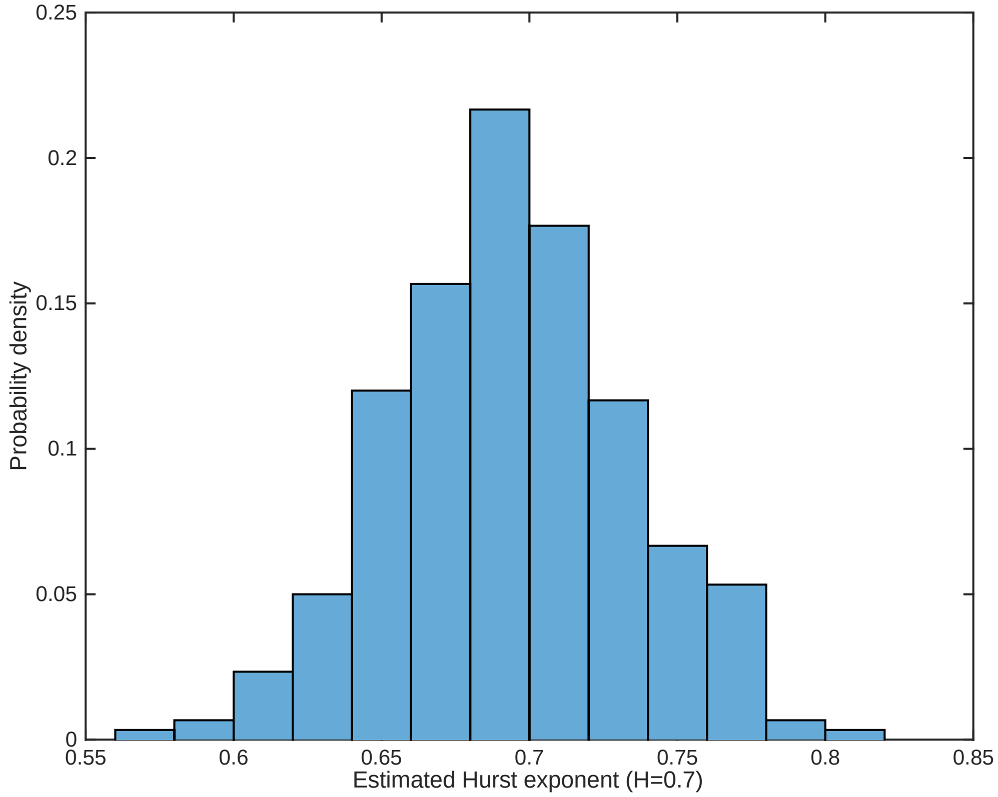

Supplementary Figure 4. Probability density of measured H from the simulation. For estimating the uncertainty in scaling exponents in short realisations of known processes, we generate 300 time series of the (monofractal) fractional brownian motion (FBM) with Hurst exponent H=0.7. Each series is $N=2^{16}=65536$ points long. This is comparable to the resolution of the NGRIP Holocene $d^{18}O$ temperature proxy. Shown is the probability density for the H exponent estimated from a best linear fit in log(F(s)) vs. log(s) for 300 realisations of the fractional brownian motion with H=0.7.

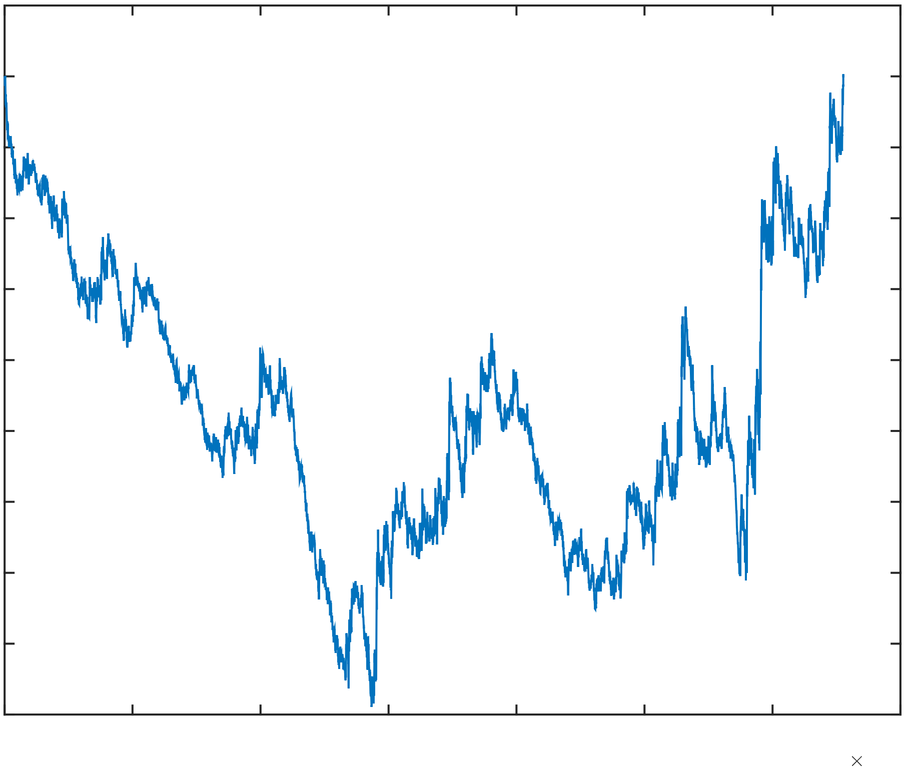

Supplementary Figure 5. Mono- vs. multifractal scaling. A realization of the constructed multifractal p-model [1] with p=0.375 used to test the detection of multifractal scaling in the finite size records.

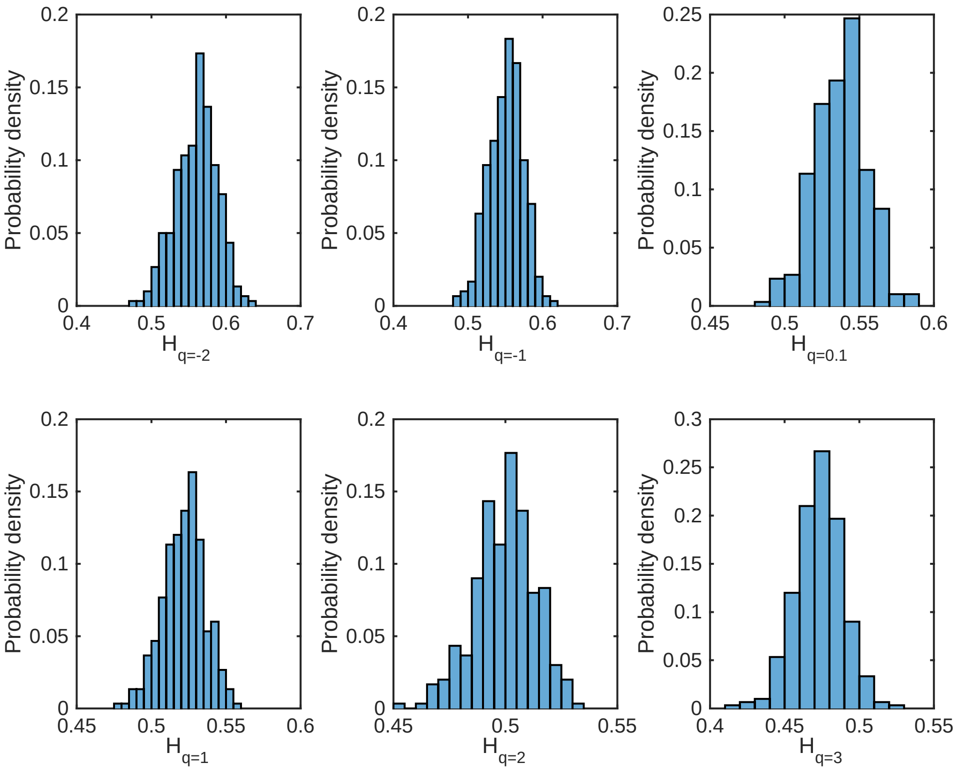

Supplementary Figure 6. Testing for multifractal scaling. In order to estimate to which extend it is possible from a time series of the length of the Holocene record to distinguish between a monofractal and a (weakly) multifractal process we have repeated the analysis above using the multifractal p-model [1]. Shown are the probability densities for the H_q exponent estimated from a best linear fit in log(F_q(s) vs. log(s) for 300 realisations of the multifractal p-model with p=0.375.

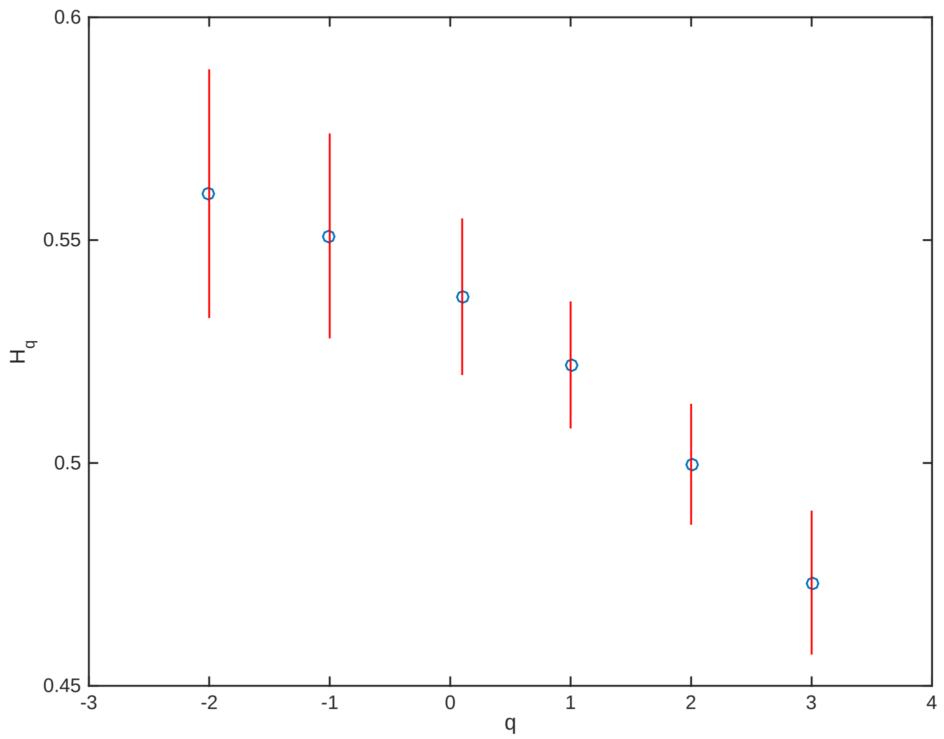

Supplementary Figure 7. Errorbars for estimates of scaling exponents obtained from simulations. Circles are measurements of H_q while the red error bars are the 1sigma-level obtained from the 300 realisations ensemble of the p-model. The multifractality can be measured for the p-model with this length of record. This does not prove but supports the result that the observed Holocene record is monofractal.

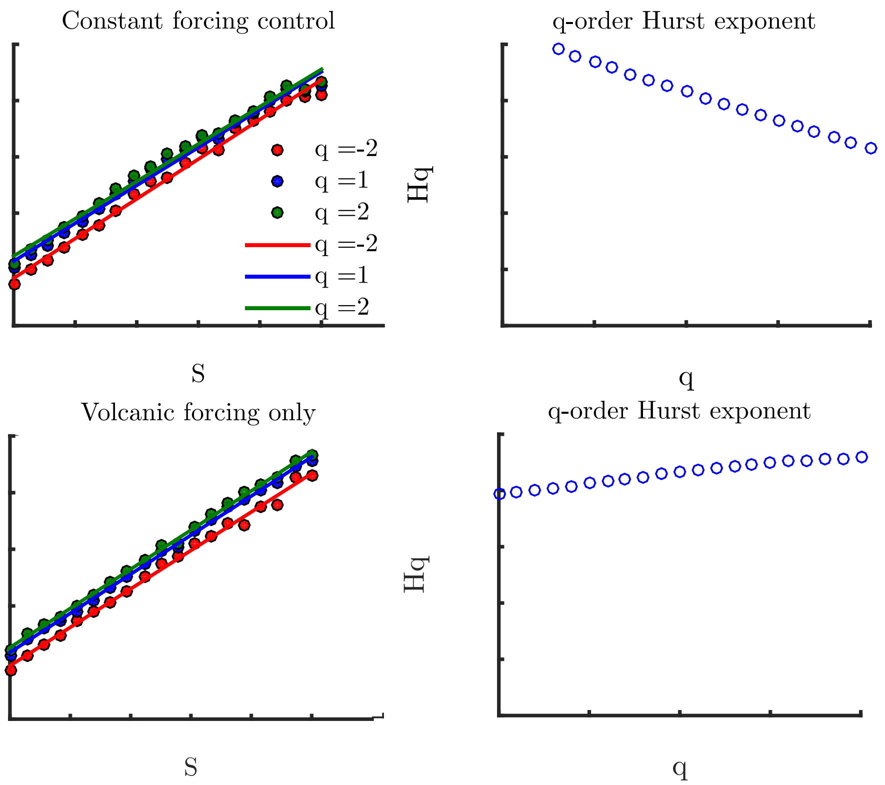

Supplementary Figure 8. Scaling in climate model simulations. We have analysed millennium simulations (850 - 2005 AD) from the CESM1-CAM5 Last Millennium Ensemble (LME) project [2]. The spectra shown are from one realisation from the Ensemble. Top panel shows the control run with constant forcing, while the bottom shows a run with volcanic forcing. The simulations are monofractal with H≈0.68 in agreement with the scaling for the NGRIP Holocene record. The simulations agree with the observed monofractal structure of the Holocene proxy record. Importantly, this result does not change between the controls run with constant forcing (top panels) and the run with volcanic forcing (bottom panels), which could in principle introduce the fractal scaling.

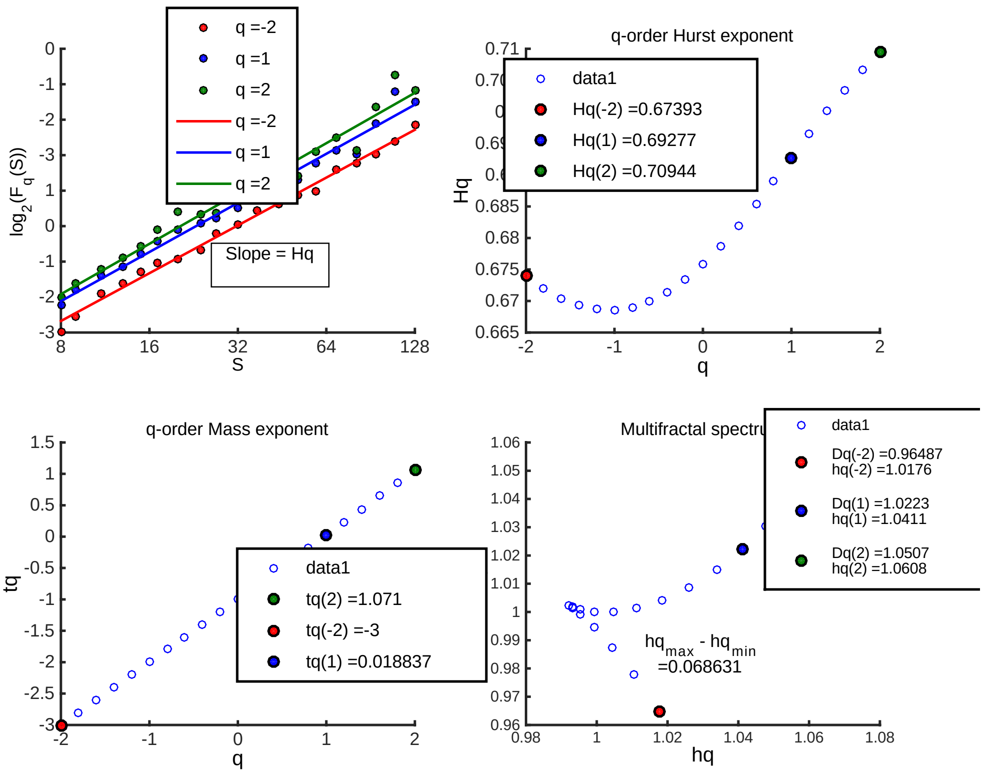

Supplementary Figure 9. Spectra for the Holocene part of the GRIP record. As a further confirmation on the robustness of the results, we have repeated the analysis on the GRIP core[3] from the Summit in Greenland. The dating of the record is done using the GICC05 time scale.

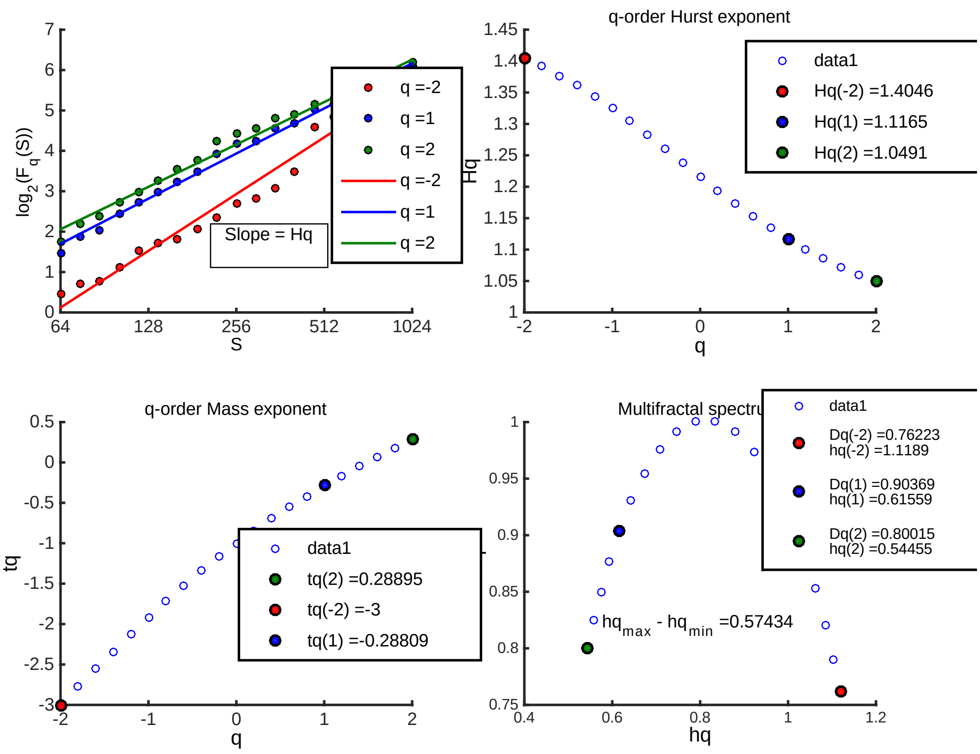

Supplementary Figure 10. Spectra for the glacial part of the GRIP record.

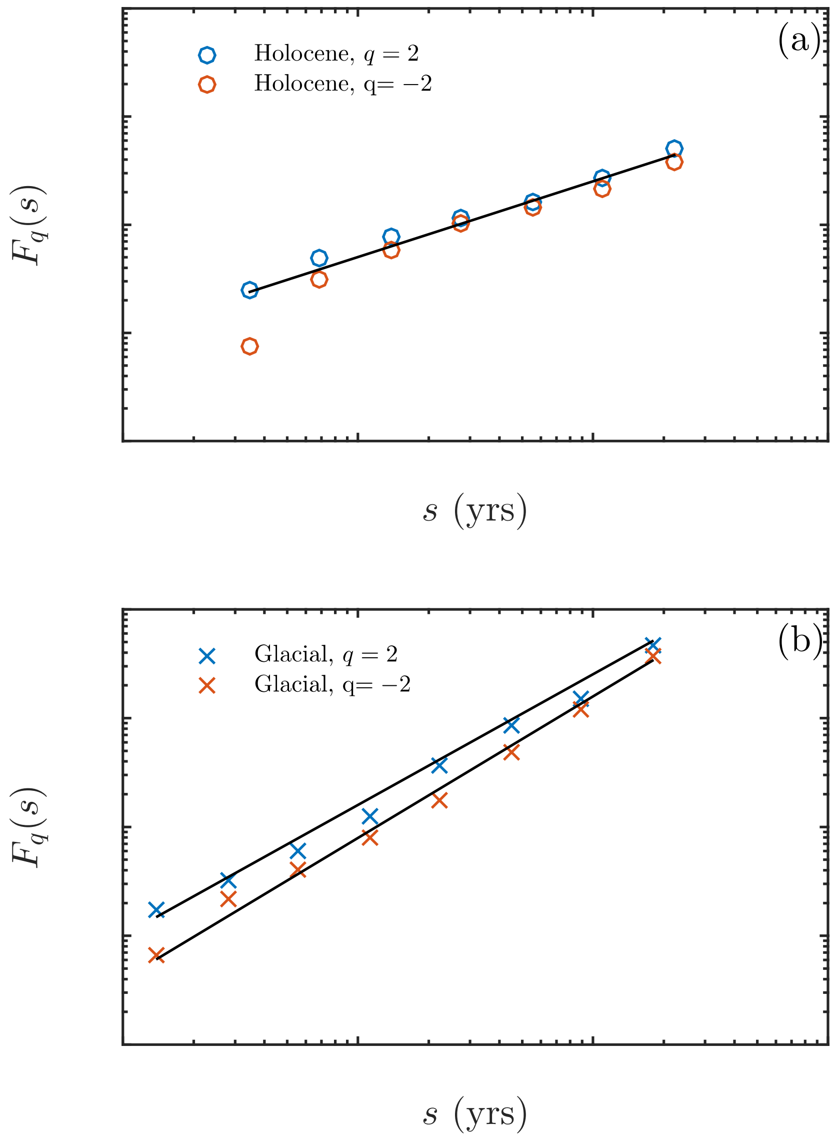

Supplementary Figure 11. EDML record. Spectra for the Holocene and glacial part of the Antarctic EDML record.

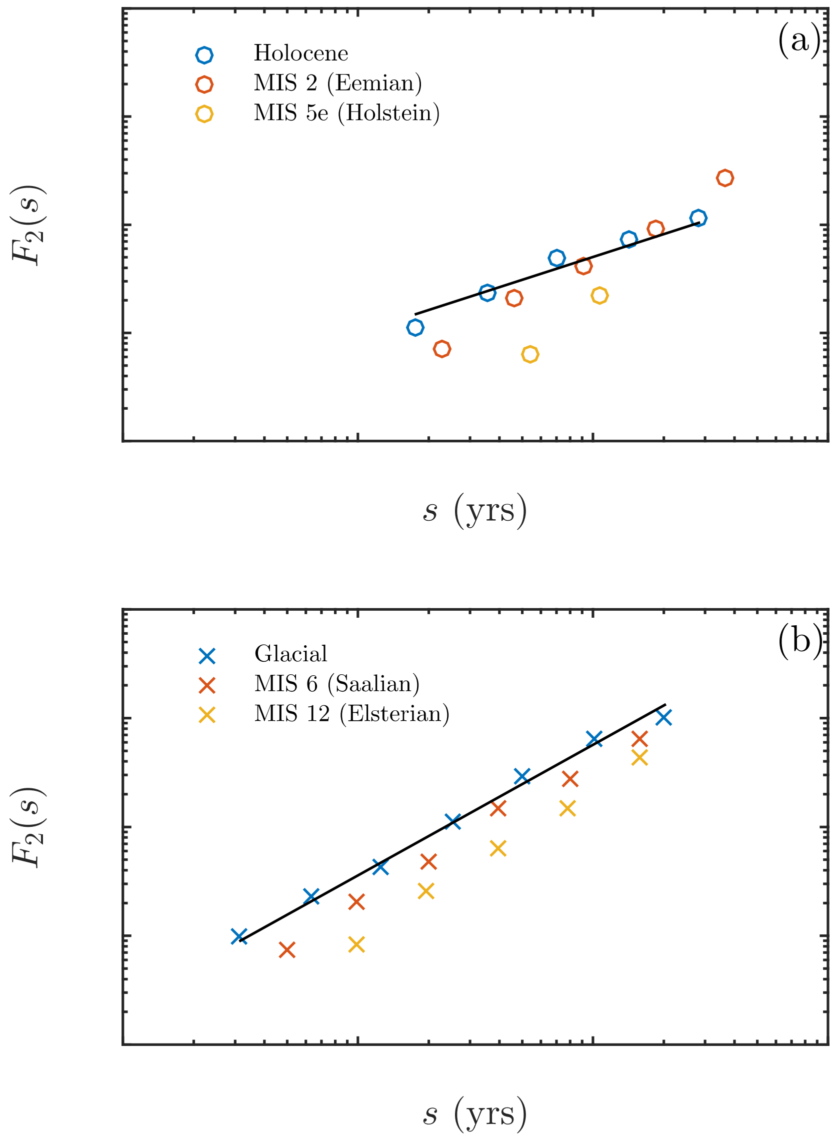

Supplementary Figure 12. Vostok record. Spectra for the last three interglacial and glacial parts of the Antarctic Vostok record.

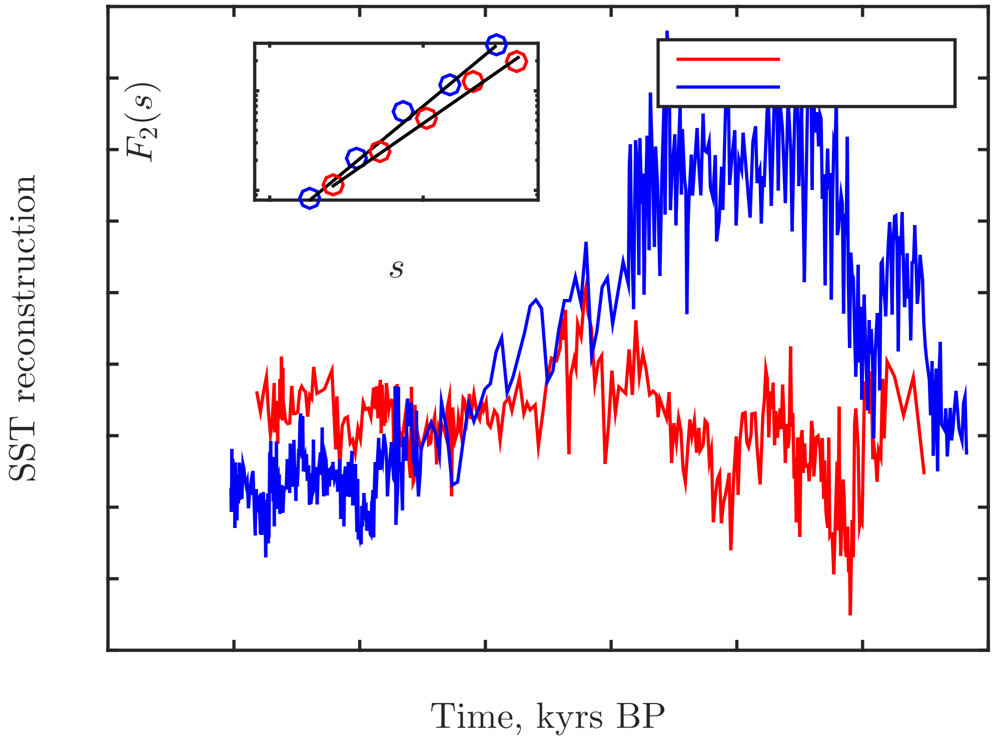

Supplementary Figure 13. Local North Atlantic sediment records. Two SST reconstructions for the Holocene in the North Atlantic. The LO09-14 core (Berner et al., 2008) is from the Reykjanes Ridge south of Iceland, while the MD95-2011 core (Berner et al., 2011) is near the Norwegian coast. The records show that the local SST variations were substantial during the Holocene and much more variable than the more extended North Atlantic climate record obtained from Greenland ice cores. The insert shows that DFA spectra with H ≈ 1.1 for LO09-14 and H ≈ 1.4 for MD95-2011. The two records are slightly anti-correlated (corr.=-0.25), which could be caused by splitting of the northward heat transport and thus indicate why the average Holocene climate represented by the Greenland ice cores is much more stable and shows a much smaller scaling exponent of H ≈ 0.7.

Supplementary References

[1] A. Davis, A. Marshak, R. Cahalan, W. Wiscombe, “Radiative smoothing in fractal clouds”, Journal of the Atmospheric Sciences 54, 241-260, (1997).

[2] B. L. Otto-Bliesner, et al., “Climate Variability and Change since 850 C.E.: An Ensemble Approach with the Community Earth System Model (CESM)”, Bulletin of the American Meteorological Society, doi: 10.1175/BAMS-D-14-00233.1, (2015).

[3] W. Dansgaard et al., “Evidence for general instability of past climate from a 250-kyr ice-core record”, Nature, 364, 218, (1993)
